# Virtual Histology of Transgenic Mouse Embryos for High-Throughput Phenotyping

**DOI:** 10.1371/journal.pgen.0020061

**Published:** 2006-04-28

**Authors:** John T Johnson, Mark S Hansen, Isabel Wu, Lindsey J Healy, Christopher R Johnson, Greg M Jones, Mario R Capecchi, Charles Keller

**Affiliations:** 1 Scientific Computing and Imaging Institute, University of Utah, Salt Lake City, Utah, United States of America; 2 Division of Pediatric Hematology-Oncology, Department of Pediatrics, University of Utah, Salt Lake City, Utah, United States of America; 3 Department of Cellular & Structural Biology and Department of Pediatrics, Children's Cancer Research Institute, The University of Texas Health Science Center, San Antonio, Texas, United States of America; 4 Department of Human Genetics, University of Utah, Salt Lake City, Utah, United States of America; 5 Howard Hughes Medical Institute, University of Utah, Salt Lake City, Utah, United States of America; The Jackson Laboratory, United States of America

## Abstract

A bold new effort to disrupt every gene in the mouse genome necessitates systematic, interdisciplinary approaches to analyzing patterning defects in the mouse embryo. We present a novel, rapid, and inexpensive method for obtaining high-resolution virtual histology for phenotypic assessment of mouse embryos. Using osmium tetroxide to differentially stain tissues followed by volumetric X-ray computed tomography to image whole embryos, isometric resolutions of 27 μm or 8 μm were achieved with scan times of 2 h or 12 h, respectively, using mid-gestation E9.5–E12.5 embryos. The datasets generated by this method are immediately amenable to state-of-the-art computational methods of organ patterning analysis. This technique to assess embryo anatomy represents a significant improvement in resolution, time, and expense for the quantitative, three-dimensional analysis of developmental patterning defects attributed to genetically engineered mutations and chemically induced embryotoxicity.

## Synopsis

Developmental biology is entering the digital age, thanks to advancements in imaging technologies, instrumentation, and software. These advancements are converging with discoveries in developmental biology to deliver unprecedented insight into how human development is impacted by the products we use, the environment we live in, and our genetic composition. Industrial societies are becoming increasingly concerned with the exposure of women and their unborn fetuses to pharmaceuticals and commonplace household chemicals. In addition, understanding genetic causes of birth defects is now possible through the isolation of specific genes, which can be efficiently disrupted in embryos, and subsequently observed for birth defects. Such studies of embryotoxicity typically involve the use of mouse embryos. However, evaluation of mouse embryos in the past has involved expensive and cumbersome external inspection and thin sectioning for view under the microscope. As such, developmental biologists have eagerly anticipated the advent of tools that would allow them to routinely assess the complex and dynamic organization of embryos using techniques that are fast and inexpensive. In this article, the authors introduce a rapid, high-quality, and inexpensive technique for the three-dimensional visualization of mouse embryos using X-ray computed tomography that is ideally suited for researchers in pharmaceutical, industrial, and academic laboratories.

## Introduction

Gene targeting in mice allows unprecedented insight into the function of genes and their roles in patterning the mammalian embryo [1]. A full understanding of mammalian development by this means, using the gene-targeting approach for every one of the approximately 25,000 or more mouse genes, may seem like a daunting task. Nevertheless, more than 10% of known mouse genes have already been disrupted by gene targeting. Moreover, the National Institutes of Health is leading an effort to create a collection of mouse lines with disruption of every known gene [2]. The challenge laid before developmental biologists will be to systemically analyze morphological phenotypes and, where possible, determine the quantitative contribution of each gene towards patterning of the embryo. Tools for this type of “phenomic” analysis must include rapid, inexpensive, and accessible high-throughput methods of high-resolution anatomical imaging (addressed here) as well as stage-specific, statistically-averaged wild-type morphological atlases that can be used to discern normal variation from mutant phenotype [3].

The introduction of magnetic resonance microscopy (MRM) represented an important time savings over classical histology in the screening of E6.5–E19 mouse embryos for mutant morphological phenotypes [4] (reviewed by Schneider and Bhattacharya [5] and Tyszka et al. [6]). However, the specialized and expensive equipment required for such high field magnetic resonance scans is not widely available. Furthermore, scans at useful resolutions (12–43 μm, but generally 25 μm) require 9–14 h of instrument time [7,8] at a cost of approximately US$200 per hour.

We introduce a new method of obtaining virtual histology using X-ray microscopic computed tomography (microCT). This technique permits mid-gestation mouse embryos to be scanned at up to 8-μm resolution in comparable or less time, and at a fraction of the expense of MRM. This new method, which employs osmium tetroxide to differentially stain tissues, will facilitate the quantitative, three-dimensional analysis of mouse developmental defects for researchers at a broad range of institutions at relatively little expense—and at resolutions and throughput comparable to or exceeding magnetic resonance methods.

## Results

### Virtual Histology by Computed Tomography Is a Powerful Method for Imaging Embryo Anatomy

**Figure 1:**
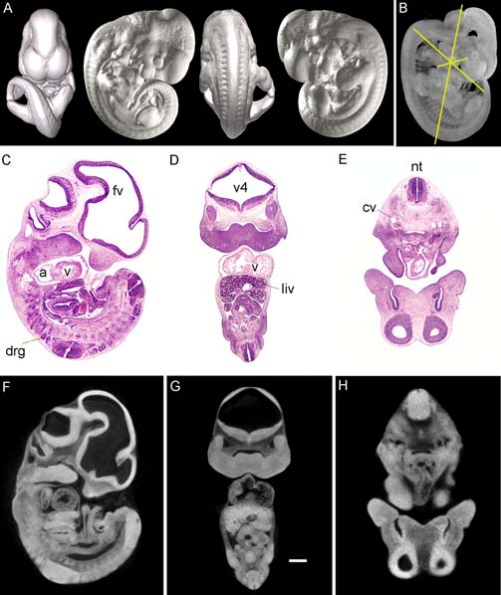
Comparison of Virtual and Paraffin Histology of an E11.5 Embryo Scanned at 8 μm (A) Isosurface renderings of the CT-scanned embryo. (B) Maximum intensity projection of the same embryo, with overlying places of section. (C–E) Sagittal, coronal, and axial sections of an E11.5 littermate. (F–H) Sagittal, coronal, and axial computed tomography sections of the embryo in panels (A) and (B), corresponding to the planes of section in panels (C–E). At low-power magnification, virtual and true paraffin histology demonstrate a similar level of detail. Scale bar indicates 400 μm. a, cardiac atrium; cv, cardinal vein; drg, dorsal root ganglia; fv, forebrain vesicle; liv, liver; nt, neural tube; v, cardiac ventricle; v4, fourth ventricle.

Formalin-fixed wild-type E11.5 embryos whose cell membranes were stained in a 1% solution of osmium tetroxide were imaged by volumetric computed tomography (CT) at 8-μm isometric resolution. External surface features of the scanned embryos were represented as isosurfaces (Figure 1A), demonstrating a level of detail comparable to a dissection microscope. Internal structures could be visualized by a semi-transparent maximum intensity projection of the entire embryo, similar to a plain radiograph (Figure 1B). In order to compare the spatial resolution of traditional optical histology to microCT-based virtual histology, paraffin-embedded 4.5-μm sections were stained with hematoxylin and eosin and visualized at 2× magnification (Figure 1C–1E). Sagittal, coronal, and axial sections by microCT-based virtual histology were comparable in the delineation of internal features (organs and tissues) to paraffin sections at the same magnification. Anatomical landmarks as small as the dorsal root ganglia, the neural tube, and the anterior cardinal vein could be easily discerned. Because osmium staining increases with lipid content of tissues, large differences in densities are observed between tissues (normalized CT attenuation values for tissue ranged from 5,785 to 32,767 Hounsfield units).

### Virtual Histology is Most Amenable to Mid-Gestation Embryos

**Figure 2:**
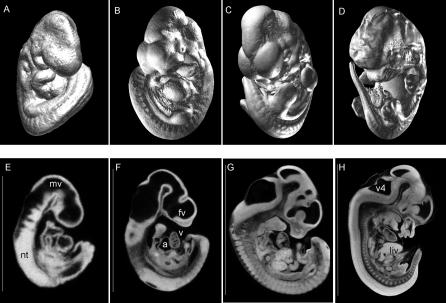
Ultra-High-Resolution Virtual Histology for a Time Series of Wild-Type Embryos (A–D) Isosurfaces of E9.5, E10.5, E11.5, and E12.5 embryos scanned at 8-μm resolution. (E–H) Corresponding sagittal sections. Vertical scale bars to the right of each embryo indicate 1.62, 2.94, 4.50, and 6.73 mm, respectively. mv, midbrain vesicle.

Unlike MRM, which does not necessarily require staining, the osmium tetroxide stain for microCT-based virtual histology is best suited to gestational ages with limited epidermal layers (unless the epidermis is manually removed with a #2 forceps or protease digested prior to staining [9]). In our experience, mid-gestation whole embryos (E8–E13.5) that lack significant epidermal development are most appropriate for this method, although skinned embryos up to E19 can be satisfactorily stained and imaged as well (unpublished data). Figure 2 demonstrates isosurfaces (Figure 2A–2D) and sagittal cross-sections (Figure 2E–2H) of a time series of E9.5, E10.5, E11.5, and E12.5 embryos, respectively, scanned at 8-μm isometric resolution. At these resolutions, features such as the developing brain vesicles, neural tube, heart chambers, and liver can be clearly delineated. Due to the increased lipid content of the liver, attenuation of osmium-stained hepatic tissue results in the highest opacity and brightness.

### Rapid 27-μm Resolution Scans Can Be Performed on a Small Animal CT Scanner

**Figure 3:**
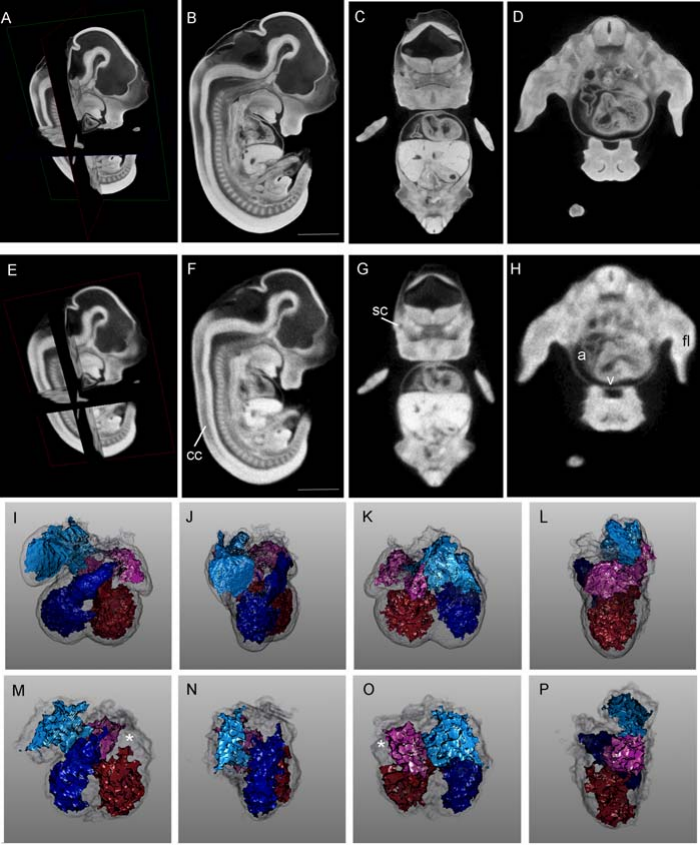
Comparison of High-Resolution (8 μm) and More Rapid (27 μm) Virtual Histology Techniques (A–D) Wild-type E12.5 embryo scanned at 8-μm resolution. (A) Orientation of the planes of section. (B) Sagittal plane. (C) Coronal plane. (D) Axial plane. (E–H) The same embryo as in (A–D), scanned by a more rapid protocol at 27-μm resolution. Most of the features present at 8-μm resolution are also appreciable at the lower 27-μm resolution. (I–P) Comparison of segmentation analysis of cardiac chambers for the 8-μm and 27-μm scans, respectively. The right atrium is teal, the right ventricle is blue, the left atrium is pink, the left ventricle is red, and the cardiac wall is transparent grey. A region of the right atrium that could not be segmented on the 27-μm scan is shown with a white asterisk (*). Scale bars in panels (B) and (F) represent 1.2 mm. Movies of sagittal, coronal, and axial planes corresponding to panels (B–D) are presented as Videos S1, S2, and S3. a, cardiac atrium; cc, central canal of the neural tube; fl, forelimb; sc, semicircular canal; v, cardiac ventricle.

Lower spatial resolutions, comparable to most typical magnetic resonance methods (25–27 μm) can be achieved with only a 2-h acquisition time on a small animal CT scanner. These live-animal scanners are more likely to be available at academic imaging core facilities. Employing a GE eXplore RS small animal scanner, we performed scans of wild-type E12.5 embryos at 27-μm isometric resolution in approximately 2 h. A comparison of 8-μm and 27-μm scans of the same embryo is shown in Figure 3. Although 8-μm sections (Figure 3A–3D) display considerably higher spatial resolution, the 27-μm sections (Figure 3E–3H) were nonetheless adequate to distinguish features such as the semicircular canal, the neural tube central canal, and the cardiac chambers. From the perspective of high-throughput phenotyping, the resolution of these 27-μm microCT scans were in the range of MRM, but at a nearly 6-fold time savings and a 300-fold cost savings. Furthermore, these 2 h, 27-μm resolution scans were adequate to perform high-quality segmentation analysis of major organ compartments, a prerequisite for computer-based, automated phenotyping (Figure 3M–3P). A caveat is that the small lumens within some organs, such as the right atrium of the heart, are less well segmented in the rapid scan than by the higher resolution scan (Figure 3I–3L). However, as shown in this figure, the same osmium-stained embryo scanned at 27-μm resolution can be scanned at 8-μm resolution when increased definition of smaller structures is necessary.

### Rapid 27-μm Scans Are Sufficient for Segmentation and Screening for Developmental Defects

**Figure 4:**
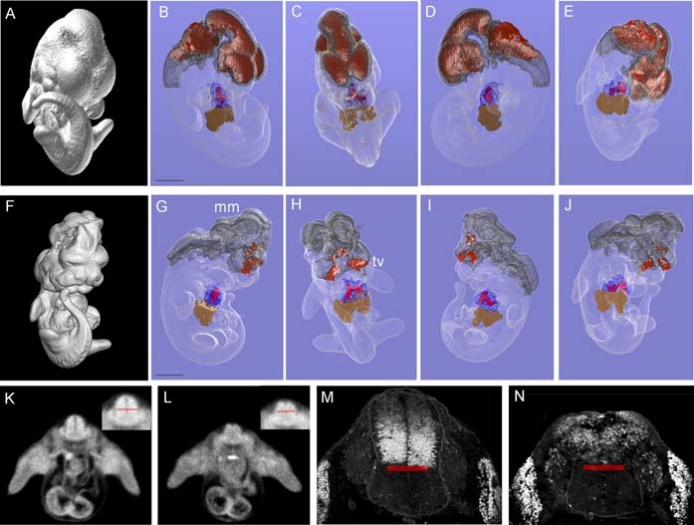
Phenotyping of Mutant Transgenic Embryos Using Segmentation Analysis Wild-type E11.5 embryo (A–E) compared to a mutant littermate (F–J) that expresses the *Pax3:Fkhr* oncogene in neural crest and myogenic tissue. (A and F) Isosurface renderings. (B–E) and (G–J) Segmentation renderings of cranial neural crest (grey), brain vesicles (red), liver (brown), cardiac muscle (blue), and the conotruncal cavity (red within the blue cardiac muscle). From the segmented renderings, the mutant embryo is noted to have partial neural tube closure failure resulting in absence of the hindbrain vesicle, overgrown midbrain mesenchyme (mm), as well as severely hypomorphic telencephalic vesicles (fv, shown in red). Movies of rotating renderings for mutant and wild-type embryos are presented as Videos S4 and S5. (K and L) MicroCT axial sections of the neural tube at the level of the forelimbs (dorsal surface is up) from wild-type and control embryos, respectively. Magnification of the neural tube is shown in the upper right insets of each panel. The sulcus limitans, which separates the dorsal and ventral neural tube, is shown as a red line. Severe blunting of the dorsal neural tube is evident in the mutant embryo. (M and N) Axial confocal microscopy sections from embryos of the same genotype and developmental stage as the corresponding axial cross-section as in panels (K and L), respectively. Sections were stained with the Pax7 immunohistochemical marker of the dorsal neural tube. Pax7-stained cells of the dorsal neural tube and flanking dermomyotome are white. The sulcus limitans is shown as a red line, confirming the dorsal neural tube blunting seen in the microCT sections. Scale bars in panels (B) and (F) are each 1.2 mm.

To test the value of microCT virtual histology for high-throughput phenotyping for major organ compartments and tissue structures of younger embryos, we utilized *Pax3:Fkhr* transgenic mouse embryos known to have complex rostral neural tube malformations [10,11]. These embryos express the *Pax3:Fkhr* fusion oncogene in place of the *Pax3* gene in the dorsal neural tube and the dermomyotome, resulting in partial failure of neural tube closure. Wild-type and *Pax3:Fkhr* mutant E11.5 embryos were scanned at 27-μm resolution, then renderings with segmentation were performed to visualize the cephalic forebrain, midbrain, and hindbrain vesicles, the heart wall and cardiac ventricles, and the liver (Figure 4A–4J). With these renderings, one can appreciate failure of neural tube closure at the level of the hindbrain and midbrain, overgrowth of the midbrain mesenchyme, as well as the hypotrophy of the telencephalic vesicles. Although these findings would have been apparent with real histology derived from paraffin-embedded specimens, the complex global three-dimensional organization of the mutant forebrain, midbrain, and hindbrain would not have been. The cardiac ventricular wall (blue) is essentially the same between wild type and mutant at this age, with no appreciable difference in the volume or patterning of the common ventricle. The liver also appears to be patterned normally in both wild type and the mutant. Using individual 27-μm planes, an additional, more subtle defect was detected in the neural tube at the level of the forelimbs (Figure 4K and 4L). Mutant embryos exhibit mispatterning (dysmorphology) of the dorsal neural tube, which was confirmed by confocal microscopy and immunohistochemical detection of the dorsal neural tube marker, *Pax7* (Figure 4M and 4N). From the point of view of a semi- or fully automated high-throughput screen for developmental patterning defects, the rapid 27-μm scan represents a feasible method of morphological typing of both complex gross and relatively subtle morphological features.

## Discussion

We present a rapid and inexpensive screening method for anatomical phenotyping of mid-gestation embryos using osmium tetroxide staining and microCT-based imaging. MicroCT-based virtual histology matches or exceeds the tissue contrast achieved by more time- and cost-intensive MRM, while delivering more than 2-fold higher resolution [3] (up to 8 μm for microCT). At lower microCT resolutions (27 μm), as many as 120 embryos can be simultaneously scanned in approximately 2 h with adequate quality for post-imaging segmentation analysis allowing the recognition of gross and subtle mutant phenotypes (Figure 4; Table 1). For increased detail of abnormalities suspected on the low-cost 27-μm scans, the same osmium-stained specimens can later be re-scanned at 8-μm resolution for unprecedented detail of organ subcompartments and fine tissue structures. We believe that this technique will be most useful as a first-line screen of embryonic defects, from which investigators could then perform traditional histological/immunohistochemical analysis of regions of interest. This technique could also be valuable in the high-throughput evaluation of teratogenic effects of medications and chemicals (e.g., embryotoxicity studies), evaluation of tissues from adult animals, and neocapillary mapping for tumor biopsies of patients undergoing anti-angiogenesis therapies. For increased throughput of these types of studies, multiple samples can be scanned simultaneously in the same field of view (e.g., six to 120 embryos can be scanned at once). This high-throughput technology may be especially applicable to Tier 1 Phenotyping in the National Institutes of Health Mouse Knockout Project [2]. The expected higher sensitivity of this embryotoxicity analysis tool also has the potential to streamline the protocols for evaluating chemical- and drug-related reproductive safety in rodents, thereby reducing the number of animals required for testing, a growing concern in the European Union and elsewhere [12].

**Table 1:**
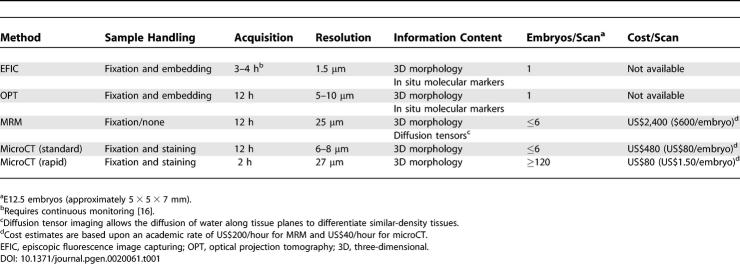
Comparison of Embryo Phenotyping Screening Methodologies and Costs

A practical consideration is whether mid-gestation is an appropriate interval for the detection of birth defects and embryotoxicity; however, in one forward genetic screen for recessive mutations, 58 of 83 (70%) of murine birth defects evaluated before and after birth had arisen prior to E13 and could be detected at E9–E13 by routine examination and microscopy [13,14]. Using microCT-based virtual histology, the sensitivity may be even higher than 70%.

We expect that an entire field of microCT-based virtual histology methods will soon emerge given the recent advances in high-resolution microCT instrumentation and the exploration of existing and new electron-dense stains. In preliminary experiments on a newly available commercially scanner, we have achieved isometric spatial resolutions as high as 6 μm in pilot studies. Our other preliminary experiments suggest that a combination of osmium and cis-platinum (or ethidium bromide) may allow for differential staining of cell membranes and nuclei, respectively, so that the staining characteristics of organs and tissues can be further differentiated (unpublished data). Furthermore, our preliminary experience and studies by Bentley (see [15]) demonstrate that osmium-stained tissue, with or without counterstains, can be later sectioned for true histological staining. The multiple uses of osmium-stained tissues will therefore speed the transition from microCT-based screens to episcopic [16] and microscopic histological verification of suspected morphological phenotypes.

In order to achieve high-throughput phenotyping, or phenomics, the computing methods for analysis must be semi-automated or fully automated. Magnetic resonance imaging of postnatal mouse brains (as well as human brains) has led to significant advances in semi- or fully automated deformable shape mapping whereby specimens of different sizes can be compared for the eventual detection of statistically different features of morphology [17]. MicroCT-based virtual histology datasets are ideally suited to this type of analysis.

MicroCT-based virtual histology is not intended to replace the generally more versatile magnetic resonance methods (for a review of MRM, see references [3,5,7]), but is instead a useful adjunct for anatomical imaging. A comparison of CT and magnetic resonance methods, applications, and costs are given in Table 1. Included for comparison are the low-throughput but exceptional resolution methods of episcopic fluorescence image capturing [16,18] and optical projection tomography [19]. Magnetic resonance imaging allows the analysis of a variety of tissue properties to be interrogated, depending upon the pulse sequence, and post-processing parameters such as diffusion tensors [17] allow the organization of tissues to visualized and modeled in unprecedented ways. Instead, microCT-based virtual histology offers a potentially higher resolution mode of morphometrics that is simple to implement, relatively inexpensive, and more rapid than comparable methods of phenotyping embryo anatomy.

## Materials and Methods

### Sample preparation.

The *Pax3:Fkhr* transgenic mice have been previously described [10,11]. Embryos were harvested at E9.5–E12.5 gestational ages, then fixed in 10% buffered formalin overnight at 4 °C. Hematoxylin and eosin–stained paraffin sections were prepared using established methods [10], then visualized at 2× magnification on a Nikon Eclipse 80i microscope (Nikon, Tokyo, Japan). Immunohistochemistry with the Pax7 monoclonal antibody (Developmental Hybridoma Studies Bank, Iowa City, Iowa, United States) was performed as previously described [10]. For microCT-based virtual histology, formalin-fixed embryos were stained using a beta version of a commercially available kit (Numira Biosciences, San Antonio, Texas, United States http://www.numirabio.com). In brief, embryos were stained to saturation overnight in a solution of 0.1 M sodium cacodylate (pH 7.2), 1% glutaraldehyde, and 1% osmium tetroxide rocking at room temperature. We note that osmium tetroxide requires careful handling that is possible in most molecular biology laboratories (for the international safety and handling information, see http://www.cdc.gov/niosh/ipcsneng/neng0528.html). Embryos were then washed for 30 min in 0.1 M sodium cacodylate buffer, and twice more for 30 min in phosphate-buffered saline. Samples were then transitioned by a series of gradients to 100% ethanol prior to scanning. For each 25%, 50%, 75%, and 100% ethanol gradient equilibration, embryos were incubated for 15–30 min, or until the embryos lost buoyancy. Prior to imaging, embryos were placed in 2-ml polypropylene microfuge tubes.

### Imaging.

High-resolution volumetric CT of embryos was performed at 8-μm^3^ isometric voxel resolution using an eXplore Locus SP MicroCT specimen scanner (GE Healthcare, London, Ontario, Canada). This volumetric scanner employs a 3,500 × 1,750 CCD (charge-coupled device) detector for Feldkamp cone-beam reconstruction, and is similar in performance to other commercially available in vitro scanners under $300,000 that are commonly operated at regional core facilities (http://ccri.uthscsa.edu/ImagingFacility.html). In this study, the platform-independent parameters of current, voltage, and exposure time were kept constant at 100 μA, 80 kVP, and 4,000 ms, respectively. For each scan, 900 evenly spaced views were averaged from eight frames/view, filtered by 0.2-mm aluminum. At 8-μm resolution, the field of view of this instrument is 15 × 15 × 15 mm. Each scan took approximately 12 h. Cost of this method is approximate US$40 per hour (US$480 per scan). Images were reconstructed with the manufacturer's proprietary EVSBeam software.

More rapid volumetric CT scans of embryos performed at 27-μm^3^ isometric voxel resolution using an eXplore Locus RS small animal MicroCT scanner (GE Healthcare). Like the specimen scanner, this live-animal volumetric scanner employs a 3,500 × 1,750 CCD detector for Feldkamp cone-beam reconstruction and is similar in performance to other commercially available in vivo scanners under $300,000 that are commonly operated at regional core facilities (http://ccri.uthscsa.edu/ImagingFacility.html). In this study, the platform-independent parameters of current, voltage, and exposure time were kept constant at 450 mA, 80 kVP, and 2000 ms, respectively. For each scan, 450 evenly spaced views were averaged from six frames/view. At 27-μm resolution, the field of view of this instrument is 45 × 45 × 45 mm. Each scan took 2 h and 4 min. Cost of this method is also approximate US$40 per hour (US$80 per scan). Images were reconstructed with the manufacturer's proprietary EVSBeam software. The 600 MB three-dimensional dataset and the raw data for the scan were transferred to a single DVD (approximate total, 4.5 GB). Preliminary visualizations (unpublished data) and virtual histology sections were generated in real time with the freely-available MicroView program (http://microview.sf.net).

### Isosurface generation.

Isosurfaces renderings of the CT datasets were generated using a combination of the open-source Teem utilities [20] and the open-source, platform-independent BioImage [21] rendering program. Both sets of software are available online (http://software.sci.utah.edu). Sub-sampling of the microCT volumes, which are significantly larger than the available memory on current graphics cards, was also performed using the Teem libraries. The final sub-sampled volumes were 255 × 255 × 255 mm and equally spaced using a nearest neighbor approach. Pre-computing of the gradient and Hessian data was also performed using the Teem libraries. The combined process of sub-sampling and generation of gradient and Hessian data produced two data volumes, which were stored together with the original CT values in a “VGH” (value, gradient, Hessian) volume. The VGH volumes were subsequently used to create isosurfaces in real time with the BioImage software package.

### Segmentation.

Image volume segmentations were done using a Watershed algorithm [22] provided in the National Library of Medicine Insight Segmentation and Registration Toolkit (ITK; http://www.itk.org). Watershed segmentation is a region-growing algorithm in which user-defined seed points are positioned in areas of interest, and a statistical analysis is made of the gradient magnitude in the local neighborhood to find the standard deviation. The structure is then found as the perimeters of the seed points extend through the continuous surrounding area, without crossing points where the gradient magnitude is beyond one standard deviation of the mean. The resulting structures are saved to disk as separate volumes. Segmentations were done on a computer equipped with 2.5 GHz Intel Pentium III processor and 2 GB of RAM. The resulting volumes were then combined into one volume by marking each segmentation as a unique value, and then merging it into the original again, such that the original value of existing segmentation is overwriten with the new marked value.

### Volume renderings.

The volume renderings of wild-type and *Pax3:Fkhr* mutant embryo datasets were created using direct volume rendering techniques using the in-house software package, Nenners [23]. Nenners reads a volume and casts *n* ^2^ geometric rays through the volume per pixel. For each ray that intersects with the volume, equally spaced samples of length Δ*t* are acquired along the respective ray. To ensure that renderings capture the fine detail of these high-resolution datasets Δ*t*≤‖*v*_*i*_ − *v*_*i* − 1_‖, where *v_i_* is the position of *i* ^th^ voxel,the data are sampled at least once per cubic voxel. Samples are acquired by convolution using separable kernels (page 197 in [24]). Cubic B-splines were used to interpolate both the CT value and gradient [25]. Color and opacity were defined at each sample point using a two-dimensional transfer function, with gradient magnitude and X-ray density defining the domain [26,27]. This was done in a front-to-back fashion, such that when the opacity of a ray becomes equal to or greater than 1, the ray is terminated early and the next ray is computed. Bounding volumes were also used to speed this process such that rays that have not yet or will not hit the volume were not sampled. The gradient was also used to approximate the Phong shading models used [28]. For Figure 3, a curvature-based transfer function [28] and depth queuing was also applied to the volume renderings. The transfer function is based on the dot product of the angle of an incident ray with the surface normal. If the dot product was less than some constant k, 0 < k < 1, then the surface was marked black to emphasis surfaces perpendicular to the viewing direction. Furthermore, a depth queue to shade interpolated samples was used to give a greater feeling of depth and to seperate foreground from background. Parameters of camera position, viewing direction, and lighting relative to the specimen were also passed to define the view of the rendering. The software was run on an SGI Onyx 3800 (Silicon Graphics, Mountain View, California, United States) at 80 s per rendering.

## Supporting Information

Video S1 Sagittal Sections of a Wild-Type E12.5 Embryo at 8-μm Resolution (1.2 MB WMV)

Video S2 Coronal Sections of a Wild-Type E12.5 Embryo at 8-μm Resolution (1.3 MB WMV)

Video S3 Axial Sections of a Wild-Type E12.5 Embryo at 8-μm Resolution (1.7 MB WMV)

Video S4 Rendering of a Segmented *Pax3:Fkhr* Mutant E11.5 Embryo This embryo corresponds to Figure 4F–4J.
(3.6 MB MOV)

Video S5 Rendering of a Segmented Wild-Type E11.5 Embryo This embryo corresponds to Figure 4A–4E.
(3.6 MB MOV)

### Accession Numbers

The National Center for Biotechnology Information (NCBI) (http://www.ncbi.nlm.nih.gov) accession number for the *Pax3:Fkhr* fusion oncogene is AY743239, and for *Pax7* is NM_011039.

## Abbreviations

CT: computed tomography
microCT: microscopic computed tomography
MRM: magnetic resonance microscopy
